# Noncoherent Decision Fusion over Fading Hybrid MACs in Wireless Sensor Networks

**DOI:** 10.3390/s19010120

**Published:** 2019-01-01

**Authors:** Shoujun Liu, Kehao Wang, Kezhong Liu, Wei Chen

**Affiliations:** 1 School of Information Engineering, Wuhan University of Technology, Wuhan 430070, China; lsj@whut.edu.cn (S.L.); kehao.wang@whut.edu.cn (K.W.); 2 School of Navigation, Wuhan University of Technology, Wuhan 430063, China; kzliu@whut.edu.cn

**Keywords:** decision fusion, distributed detection, hybrid MAC, wireless sensor networks

## Abstract

In this paper, we consider the problem of decision fusion for noncoherent detection in a wireless sensor network. Novel to the current work is the integration of the hybrid multi-access channel (MAC) in the fusion rule design. We assume that sensors transmit their local binary decisions over a hybrid MAC which is a composite of conventional orthogonal and nonorthogonal MACs. Under Rayleigh fading scenario, we present a likelihood ratio (LR)-based fusion rule, which has been shown to be optimal through theoretical analysis and simulation. However, it requires a large amount of computation, which is not easily implemented in resource-constrained sensor networks. Therefore, three sub-optimal alternatives with low-complexity are proposed, namely the weighed energy detector (WED), the deflection-coefficient-maximization (DCM), and the two-step (TS) rules. We show that when the channel signal-to-noise ratio (SNR) is low, the LR-based fusion rule reduces to the WED rule; at high-channel SNR, it is equivalent to the TS rule; and at moderate-channel SNR, it can be approached closely by the DCM rule. Compared with the conventional orthogonal and nonorthogonal MACs, numerical results show that the hybrid MAC with the proposed fusion rules can improve the detection performance when the channel SNR is medium.

## 1. Introduction

Wireless sensor networks (WSNs) consist of a large number of geographically distributed sensors that have limited resources, such as energy, processing capabilities, and communication bandwidth [1]. With sensor collaboration, potentially powerful networks can be constructed to accomplish certain tasks, such as environment monitoring, surveillance, health care, and home automation [2,3,4,5,6,7,8,9].

A prevailing model used for such applications is the orthogonal multi-access channel (MAC) model wherein sensors make local decisions based on their observations and subsequently transmit these decisions in parallel to a fusion center (FC). Numerous researchers have focused on the problem of distributed detection (decision fusion) over orthogonal MACs [10,11,12,13,14,15,16,17,18,19,20,21].

Another type of MACs from sensors to the FC is the nonorthogonal MAC [22]. In this case, multiple sensors are allowed to communicate with an FC through the same channel. Using this scheme, bandwidth requirement or detection delay can be significantly reduced. However, in order to realize the nonorthogonal MAC, channel information needs to be fed back from the FC to each sensor so that synchronization among all the sensors can be established. Under the assumption of perfect synchronization, the type-based multiple-access scheme is studied in [23,24,25]. In [26], it is shown that the nonorthogonal MAC can achieve the same asymptotic performance as centralized detection. When wireless channels are subject to fading, in [27,28], the optimal likelihood ratio (LR) fusion rule is shown to be equivalent to a simple energy test under both Rayleigh and Rician fading channels. Further, decision fusion for WSNs with space diversity has been studied in [29,30,31,32,33]. Obviously, space diversity can effectively suppress the channel fading and hence lead to good performance. However, to employ space diversity, we need multiple antennas to be equipped at the FC. Due to the size constraint of sensors, this could be impractical for many WSN applications [34]. An alternative for space diversity is the cooperative transmission scheme [35,36], in which multiple relays are used to assist the sensors to send their decisions to the FC.

Recently, the idea of hybrid MACs with sensor grouping has emerged in [37]. For hybrid MACs, the orthogonal MAC is adopted across different groups, whereas the nonorthogonal MAC is used for sensors within each group. A flexible trade-off between these two MAC schemes can therefore be obtained by changing the number of groups and the number of sensors in each group. Using coherent detection and assuming channel gains are available, the hybrid MAC is shown to provide more performance choices than orthogonal and nonorthogonal MACs [38]. The use of coherent detection requires the knowledge of channel phrase. However, acquiring this phrase information usually results in additional training overhead, which may be too costly for resource-constrained sensor networks. Moreover, due to limited resource, the assumption that channel gains are known at the fusion center may be too strong. Thus, in this paper, we consider the case of noncoherent detection in the hybrid MAC WSN. Our goal here is to develop fusion rules which require only the knowledge of channel statistics instead of channel gains. Specifically, we present the optimal fusion rule based on the LR test and derive three suboptimal alternatives with low complexity, namely the weighed energy detector (WED), the deflection-coefficient-maximization (DCM), and the two-step (TS) rules.

The remainder of the paper is organized as follows. In Section 2, we formulate the fusion problem in the hybrid MAC WSN. In Section 3, we derive the optimal LR-based fusion rule and three sub-optimal alternatives. Performance analysis is contained in Section 4. Finally, some concluding remarks and future work are presented in Section 5.

*Notation*—Lower-case bold letters denote vectors; E[⋅], Var[⋅], D[⋅], and ${\left(\cdot \right)}^{T}$ are used to denote expectation, variance, deflection coefficient, and transpose, respectively; P(⋅), p(⋅) denote probability mass functions and probability density functions (pdf), in particular P(A|B) and p(a|b) represent the probability of event *A* conditioned on event *B* and the pdf of random variable *a* conditioned on random variable *b*, respectively.

## 2. System Model

**Figure 1:**
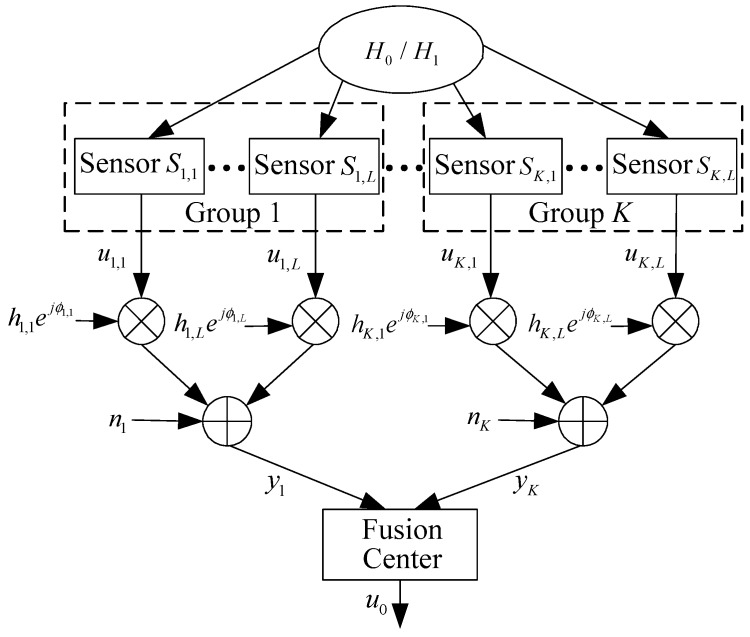
System model.

Consider a sensor network with *N* sensors, where each sensor collects its observation generated according to either $H_{0}$ (e.g., the target is absent) or $H_{1}$ (e.g., the target is present), which are two hypotheses being tested. The prior probabilities of $H_{0}$ and $H_{1}$ are denoted by $P(H_{0})$ and $P(H_{1})$, respectively. After receiving its observation, each sensor makes a binary decision and transmits it with ON-OFF signaling to the FC. The model of the distributed detection system is illustrated in Figure 1. The *N* sensors are divided into *K* groups, with the *k*th group containing $L_{k}$ sensors, and $N=\sum_{k=1}^{K}L_{k}$. Denote $S_{k,l}$, $l=1,\ldots ,L_{k}$ as the *l*th sensor in the *k*th group, and $u_{k,l}\in \left\{0,\;1\right\}$ as the binary decision made by $S_{k,l}$. For sensor $S_{k,l}$, it will transmit a pulse (i.e., $u_{k,l}=1$) to the FC if $H_{1}$ is decided, and will remain silent (i.e., $u_{k,l}=0$) during its transmission period if $H_{0}$ is decided. The detection performance of sensor $S_{k,l}$ can be characterized by its detection probability:

(1) $$P_{d_{k,l}}=P(u_{k,l}=1|H_{1})$$

and its false alarm probability:

(2) $$P_{f_{k,l}}=P(u_{k,l}=1|H_{0}).$$

In hybrid MAC networks, sensors in each group transmit their decisions via the same channel (i.e., the same time slot or frequency band) and different groups communicate with the FC through independent and mutually orthogonal channels. Thus, the received signal at the FC can be expressed as

(3) $$y_{k}=\sum_{l=1}^{L_{k}}h_{k,l}e^{j\phi_{k,l}}u_{k,l}+n_{k},\;k=1,\ldots ,K\;,$$

where $h_{k,l}$ is the real-valued channel gain, $\phi_{k,l}$ is the channel phase, and $n_{k}$ is zero-mean complex Gaussian noise with variance $2\sigma_{k}^{2}$.

## 3. Noncoherent Decision Fusion

Noncoherent reception is a useful technique because it does not require the knowledge of channel phase. Using such technique, the FC can employ fusion rules based on the received signal envelop, or equivalently, the signal power. Therefore, we consider the case of noncoherent reception at the FC and develop fusion rules based on the received signal power $\left\{z_{k}={\left|y_{k}\right|}^{2},\;k=1,\ldots ,K\right\}$.

### 3.1. LR-Based Fusion Rule

The optimum fusion rule can be formulated based on the log-likelihood ratio test:

(4) $$\Lambda_{LR}=\ln\frac{p(z_{1},\ldots ,z_{K}|H_{1})}{p(z_{1},\ldots ,z_{K}|H_{0})}\overset{H_{1}}{\underset{H_{0}}{≷}}\gamma ,$$

where the threshold γ can be determined from the false alarm constraint in the Neyman–Pearson test or can be chosen to minimize the fusion error probability in the Bayesian test [39].

Define a random variable $M_{k}≜M(u_{k})=\sum_{l=1}^{L_{k}}u_{k,l}$, where $u_{k}={\left(u_{k,1},u_{k,2},\cdots ,u_{k,L_{k}}\right)}^{T}$. Assuming a Rayleigh fading channel with unit power, we can obtain [27]

(5) $$p\left(z_{k}|M_{k}=m\right)=\frac{1}{m+2\sigma_{k}^{2}}e^{-\frac{z_{k}}{m+2\sigma_{k}^{2}}}.$$

Subsequently, we have

(6) $$p\left(z_{k}|H_{i}\right)=\sum_{m=0}^{L_{k}}p\left(z_{k}|M_{k}=m\right)P\left(M_{k}=m|H_{i}\right),\;i=0,1,$$

where $P\left(M_{k}=m|H_{i}\right)$ can be represented by the Poisson-binomial distribution, namely

(7) $$P\left(M_{k}=m|H_{0}\right)=\sum_{u_{k}:M(u_{k})=m}\prod_{l=1}^{L_{k}}{\left(P_{f_{k,l}}\right)}^{u_{k,l}}\prod_{s=1}^{L_{k}}{\left(1-P_{f_{k,s}}\right)}^{1-u_{k,s}},$$

(8) $$P\left(M_{k}=m|H_{1}\right)=\sum_{u_{k}:M(u_{k})=m}\prod_{l=1}^{L_{k}}{\left(P_{d_{k,l}}\right)}^{u_{k,l}}\prod_{s=1}^{L_{k}}{\left(1-P_{d_{k,s}}\right)}^{1-u_{k,s}}.$$

Further, if local decisions are independent and identically distributed (i.i.d.), i.e., $P_{f_{k,l}}=P_{f}$ and $P_{d_{k,l}}=P_{d}$, we have

(9) $$P\left(M_{k}=m|H_{0}\right)=\left(\begin{array}{l} L_{k}\\ m \end{array}\right){\left(P_{f}\right)}^{m}{\left(1-P_{f}\right)}^{L_{k}-m},$$

(10) $$P\left(M_{k}=m|H_{1}\right)=\left(\begin{array}{l} L_{k}\\ m \end{array}\right){\left(P_{d}\right)}^{m}{\left(1-P_{d}\right)}^{L_{k}-m}.$$

Substituting (5), (9) and (10) into (6), and using (4), we can obtain the optimal LR-based fusion statistic:

(11) $$\begin{array}{ll} \Lambda_{LR} & =\ln\frac{p(z_{1},z_{2},\cdots ,z_{K}|H_{1})}{p(z_{1},z_{2},\cdots ,z_{K}|H_{0})}\\ & =\sum_{k=1}^{K}\ln\frac{\sum_{m=0}^{L_{k}}\left(\begin{array}{l} L_{k}\\ m \end{array}\right)\frac{{\left(P_{d}\right)}^{m}{\left(1-P_{d}\right)}^{L_{k}-m}}{m+2\sigma_{k}^{2}}e^{-\frac{z_{k}}{m+2\sigma_{k}^{2}}}}{\sum_{m=0}^{L_{k}}\left(\begin{array}{l} L_{k}\\ m \end{array}\right)\frac{{\left(P_{f}\right)}^{m}{\left(1-P_{f}\right)}^{L_{k}-m}}{m+2\sigma_{k}^{2}}e^{-\frac{z_{k}}{m+2\sigma_{k}^{2}}}} \end{array}$$

where the assumption of conditional independence of $z_{k},\;k=1,\ldots ,K$, is used.

We can see that, as the number of groups increases, the test statistic in Equation (11) via sums of exponential functions becomes more and more complicated. Since most WSNs are resource constrained with regards to energy and processing capabilities, the optimal LR-based fusion rule with high complexity may be unsuitable for many practical WSN applications. Suboptimal alternatives with reduced complexity are then more desirable.

### 3.2. WED Fusion Rule

In this part, we consider the low-SNR approximation for the LR-based fusion rule and have the following proposition.

**Proposition** **1.** *As the channel noise variance*$\sigma_{k}^{2}\to \infty$, *the LR-based fusion statistic in Equation (11) reduces to a form analogous to a WED statistic:*

(12) $$\Lambda_{WED}=\sum_{k=1}^{K}\sum_{m=0}^{L_{k}}\left(\begin{array}{l} L_{k}\\ m \end{array}\right)\;\frac{{\left(P_{f}\right)}^{m}{\left(1-P_{f}\right)}^{L_{k}-m}-{\left(P_{d}\right)}^{m}{\left(1-P_{d}\right)}^{L_{k}-m}}{m+2\sigma_{k}^{2}}z_{k}.$$

**Proof.**  For low SNR, i.e., $\sigma_{k}^{2}\to \infty$,

$$\begin{array}{ll} \Lambda_{LR}\;\approx & \sum_{k=1}^{K}\ln\frac{\sum_{m=0}^{L_{k}}\left(\begin{array}{l} L_{k}\\ m \end{array}\right){\left(P_{d}\right)}^{m}{\left(1-P_{d}\right)}^{L_{k}-m}e^{-\frac{z_{k}}{m+2\sigma_{k}^{2}}}}{\sum_{m=0}^{L_{k}}\left(\begin{array}{l} L_{k}\\ m \end{array}\right){\left(P_{f}\right)}^{m}{\left(1-P_{f}\right)}^{L_{k}-m}e^{-\frac{z_{k}}{m+2\sigma_{k}^{2}}}}\\ & \overset{\left(a\right)}{\approx }\sum_{k=1}^{K}\ln\frac{\sum_{m=0}^{L_{k}}\left(\begin{array}{l} L_{k}\\ m \end{array}\right){\left(P_{d}\right)}^{m}{\left(1-P_{d}\right)}^{L_{k}-m}(1-\frac{z_{k}}{m+2\sigma_{k}^{2}})}{\sum_{m=0}^{L_{k}}\left(\begin{array}{l} L_{k}\\ m \end{array}\right){\left(P_{f}\right)}^{m}{\left(1-P_{f}\right)}^{L_{k}-m}(1-\frac{z_{k}}{m+2\sigma_{k}^{2}})}\\ & =\sum_{k=1}^{K}\ln\frac{1-\sum_{m=0}^{L_{k}}\left(\begin{array}{l} L_{k}\\ m \end{array}\right){\left(P_{d}\right)}^{m}{\left(1-P_{d}\right)}^{L_{k}-m}\frac{z_{k}}{m+2\sigma_{k}^{2}}}{1-\sum_{m=0}^{L_{k}}\left(\begin{array}{l} L_{k}\\ m \end{array}\right){\left(P_{f}\right)}^{m}{\left(1-P_{f}\right)}^{L_{k}-m}\frac{z_{k}}{m+2\sigma_{k}^{2}}}\\ & \overset{\left(b\right)}{\approx }\sum_{k=1}^{K}\sum_{m=0}^{L_{k}}\left(\begin{array}{l} L_{k}\\ m \end{array}\right)\left[{\left(P_{f}\right)}^{m}{\left(1-P_{f}\right)}^{L_{k}-m}-{\left(P_{d}\right)}^{m}{\left(1-P_{d}\right)}^{L_{k}-m}\right]\frac{z_{k}}{m+2\sigma_{k}^{2}} \end{array}$$

where we have used $e^{x}\approx 1+x$ for small *x* in (*a*) and ln(1+x)≈x for small *x* in (*b*). □

Notice that the WED fusion statistic in Equation (12) is a linear combination of the received powers $z_{k}\prime s$. Under the condition that sensors are uniformly grouped and all the channels have the same noise power, i.e., $L_{k}=L$ and $\sigma_{k}^{2}=\sigma^{2}$ for all *k*, the WED fusion statistic can reduce to a form analogous to a simple ED statistic:

(13) $$\Lambda_{ED}=\sum_{k=1}^{K}z_{k}.$$

### 3.3. Decision Fusion Rule via Maximization of Deflection Coefficient

Different from Section 3.2, in this part, we will obtain the combining weights of the linear-combining fusion statistic by maximizing the deflection coefficient [11]. The deflection coefficient could reflect the output SNR and is often used to characterize the performance of a binary hypothesis test. It is worth noting that the use of deflection criterion usually yields a robust performance in many detection problems [12]. The deflection coefficient is defined as

(14) $$D(\Lambda )=\frac{[E(\Lambda |H_{1})-E(\Lambda |H_{0}){]}^{2}}{\mathrm{Var}(\Lambda |H_{0})},$$

where E (⋅) and Var (⋅) denote the mean and variance of the fusion statistic, respectively. Using Equation (14), we have the following proposition.

**Proposition** **2.** *Under the criterion of maximizing the deflection coefficient, the linear-combining fusion statistic, referred to as the DCM statistic, is given by*

(15) $$\Lambda_{DCM}=\sum_{k=1}^{K}\frac{L_{k}z_{k}}{L_{k}^{2}P_{f}^{2}+L_{k}\left[(2+4\sigma_{k}^{2})P_{f}-2P_{f}^{2}\right]+4\sigma_{k}^{4}}.$$

**Proof.**  See the Appendix A. □

It is clear that when sensors are uniformly grouped and all the channels have the same noise power, the DCM fusion statistic is equivalent to the ED statistic. Thus, the DCM and WED fusion statistics coincide in this special case. It is worth noting that the DCM fusion statistic only requires the mean and the variance, which makes it very convenient to use for many practical applications.

### 3.4. Two-Step Fusion Rule

Motivated by the decode-then-fuse approach proposed in Equation [29], we now consider the case of high-channel SNR and develop the two-step fusion statistic as follows.

In the first step, each group makes a binary decision ${\tilde{u}}_{k}$ based on $z_{k}$. This is equivalent to a distributed detection using the nonorthogonal MAC, and the decision ${\tilde{u}}_{k}$ can be obtained through the maximum likelihood (ML) detector [39] as

(16) $$p(z_{k}|H_{1})\overset{{\tilde{u}}_{k}=1}{\underset{{\tilde{u}}_{k}=0}{≷}}p(z_{k}|H_{0}).$$

As shown in Equation [28], this ML detector is equivalent to a simple threshold test on $z_{k}$. Therefore, we have

(17) $${\tilde{u}}_{k}=I(z_{k}\geq \tau_{k}),$$

where I(⋅) is the indicator function, and the threshold $\tau_{k}$ of the *k*th group can be calculated by

(18) $$\sum_{m=0}^{L_{k}}\left(\begin{array}{l} L_{k}\\ m \end{array}\right)\frac{{\left(P_{f}\right)}^{m}{\left(1-P_{f}\right)}^{L_{k}-m}-{\left(P_{d}\right)}^{m}{\left(1-P_{d}\right)}^{L_{k}-m}}{m+2\sigma_{k}^{2}}e^{-\frac{\tau_{k}}{m+2\sigma_{k}^{2}}}=0.$$

Define $\tilde{u}=\left\{{\tilde{u}}_{k},k=1,\ldots ,K\right\}$ and the probabilities of false alarm and detection of the *k*th group by $P_{F_{k}}$ and $P_{D_{k}}$, respectively. Thus, we have

(19) $$\begin{array}{ll} P\left(\tilde{u}|H_{0}\right) & =\prod_{z_{k}>\tau_{k}}P\left({\tilde{u}}_{k}=1|H_{0}\right)\prod_{z_{k}<\tau_{k}}P\left({\tilde{u}}_{k}=0|H_{0}\right)\\ & =\prod_{z_{k}>\tau_{k}}P_{F_{k}}\prod_{z_{k}<\tau_{k}}(1-P_{F_{k}}) \end{array}$$

(20) $$\begin{array}{ll} P\left(\tilde{u}|H_{1}\right) & =\prod_{z_{k}>\tau_{k}}P\left({\tilde{u}}_{k}=1|H_{1}\right)\prod_{z_{k}<\tau_{k}}P\left({\tilde{u}}_{k}=0|H_{1}\right)\\ & =\prod_{z_{k}>\tau_{k}}P_{D_{k}}\prod_{z_{k}<\tau_{k}}(1-P_{D_{k}}) \end{array}$$

where $P_{F_{k}}$ and $P_{D_{k}}$ can be obtained by

(21) $$P_{F_{k}}=\sum_{m=0}^{L_{k}}\left(\begin{array}{l} L_{k}\\ m \end{array}\right){\left(P_{f}\right)}^{m}{\left(1-P_{f}\right)}^{L_{k}-m}e^{-\frac{\tau_{k}}{m+2\sigma_{k}^{2}}},$$

(22) $$P_{D_{k}}=\sum_{m=0}^{L_{k}}\left(\begin{array}{l} L_{k}\\ m \end{array}\right){\left(P_{d}\right)}^{m}{\left(1-P_{d}\right)}^{L_{k}-m}e^{-\frac{\tau_{k}}{m+2\sigma_{k}^{2}}}.$$

In the second step, the fusion center makes a global decision $u_{0}$ based on the likelihood ratio test on $\tilde{u}$:

(23) $$\Lambda_{TS}=\ln\frac{P\left(\tilde{u}|H_{1}\right)}{P\left(\tilde{u}|H_{0}\right)}=\sum_{z_{k}>\tau_{k}}\ln\frac{P_{D_{k}}}{P_{F_{k}}}+\sum_{z_{k}<\tau_{k}}\ln\frac{1-P_{D_{k}}}{1-P_{F_{k}}}.$$

Note that for high-channel SNR, all the groups make correct detection with a large likelihood and this two-step fusion statistic, referred to as the TS statistic, should be a good approximation for the optimal LR-based fusion statistic.

## 4. Performance Analysis

### 4.1. Asymptotic Behaviors of Linear-Combining Fusion Rules

In the following, we evaluate asymptotic behaviors for uniformly grouped hybrid MAC. We have shown in Section 3 that, in the case of the same channel SNR, the proposed linear-combining statistics (i.e., WED and DCM statistics) will reduce to a simple ED statistic. Thus, in this part, we focus on this ED statistic and evaluate its asymptotic behaviors as K→∞ and L→∞, respectively.

#### 4.1.1. Asymptotic Behavior as K→∞

When $L_{k}=L$ and $\sigma_{k}^{2}=\sigma^{2}$ for all *k*, the ED fusion statistic in Equation (13) is the sum of i.i.d. random variables. Therefore, if the number of groups (i.e., *K*) is large, this fusion statistic can be approximated by Gaussian distribution according to the central limit theorem. Thus, we have

(24) $$\Lambda_{ED}\left\{ \begin{array}{l} ∼Ν(\mu_{0},\sigma_{ED_{0}}^{2}),\;\mathrm{under}\;H_{0}\\ ∼Ν(\mu_{1},\sigma_{ED_{1}}^{2}),\;\mathrm{under}\;H_{1} \end{array}\right.,$$

where $X∼Ν(\mu ,\sigma_{ED}^{2})$ denotes that *X* is a Gaussian random variable with mean μ and variance $\sigma_{ED}^{2}$, and where $\mu_{i}$ and $\sigma_{ED_{i}}^{2}$, for *i* = 0,1, are given by

(25) $$\mu_{0}=NP_{f}+2K\sigma^{2},$$

(26) $$\sigma_{ED_{0}}^{2}=(L-2)NP_{f}^{2}+(2+4\sigma^{2})NP_{f}+4K\sigma^{4},$$

(27) $$\mu_{1}=NP_{d}+2K\sigma^{2},$$

(28) $$\sigma_{ED_{1}}^{2}=(L-2)NP_{d}^{2}+(2+4\sigma^{2})NP_{d}+4K\sigma^{4}.$$

#### 4.1.2. Asymptotic Behavior as L→∞

When the group size (i.e., *L*) is large, we have the following proposition.

**Proposition** **3.** *As* L→∞ *, the system-level probabilities of detection and false alarm are, respectively*

(29) $$P_{D_{0}}=e^{-\frac{\gamma }{LP_{d}+2\sigma^{2}}}\sum_{i=0}^{K-1}\frac{1}{i!}{\left(\frac{\gamma }{LP_{d}+2\sigma^{2}}\right)}^{i}$$

*and*

(30) $$P_{F_{0}}=e^{-\frac{\gamma }{LP_{f}+2\sigma^{2}}}\sum_{i=0}^{K-1}\frac{1}{i!}{\left(\frac{\gamma }{LP_{f}+2\sigma^{2}}\right)}^{i},$$

*where* γ *is a threshold.*

**Proof.**  As L→∞, the conditional probability density function of $z_{k}$ can be approximated by Ref. [27]

(31) $$p\left(z_{k}|H_{1}\right)=\frac{1}{LP_{d}+2\sigma^{2}}e^{-\frac{z_{k}}{LP_{d}+2\sigma^{2}}}\;,$$

(32) $$p\left(z_{k}|H_{0}\right)=\frac{1}{LP_{f}+2\sigma^{2}}e^{-\frac{z_{k}}{LP_{f}+2\sigma^{2}}}\;.$$

Since $\Lambda_{ED}$ in Equation (13) is the sum of random variables obeying the exponential distribution, we can obtain

(33) $$p\left(\Lambda_{ED}=x|H_{1}\right)=\frac{x^{K-1}}{(K-1)!{\left(LP_{d}+2\sigma^{2}\right)}^{K}}e^{-\frac{x}{LP_{d}+2\sigma^{2}}}\;,$$

(34) $$p\left(\Lambda_{ED}=x|H_{0}\right)=\frac{x^{K-1}}{(K-1)!{\left(LP_{f}+2\sigma^{2}\right)}^{K}}e^{-\frac{x}{LP_{f}+2\sigma^{2}}}\;.$$

With the above results, we can derive the system-level probability of detection:

(35) $$\begin{array}{ll} P_{D_{0}} & =\int_{\gamma }^{\infty }p\left(\Lambda_{ED}=x|H_{1}\right)dx\\ & =\frac{1}{(K-1)!{\left(LP_{d}+2\sigma^{2}\right)}^{K}}e^{-\frac{\gamma }{LP_{d}+2\sigma^{2}}}\sum_{i=0}^{K-1}i!\left(\begin{array}{l} K-1\\ \;i \end{array}\right)\gamma^{K-1-i}{\left(LP_{d}+2\sigma^{2}\right)}^{i+1}\;\\ & =e^{-\frac{\gamma }{LP_{d}+2\sigma^{2}}}\sum_{i=0}^{K-1}\frac{1}{i!}{\left(\frac{\gamma }{LP_{d}+2\sigma^{2}}\right)}^{i} \end{array}$$

In the above derivation, we have used the formula in Ref. ([40], Equation (2.321)). Similarly, we have the system-level false alarm rate as in Equation (30). □

### 4.2. Performance Analysis of the TS Fusion Rule

In evaluating the performance of the TS fusion rule, it is assumed that sensors are uniformly grouped and all the channels have the same noise power. The system-level probabilities of false alarm, detection, and error are provided in the following proposition.

**Proposition** **4.**

*(a)*  *For the Neyman–Pearson detection, the system-level probabilities of false alarm and detection are given by*
  (36) $$P_{F_{0}}=\sum_{i=K_{\tau }}^{K}\left(\begin{array}{l} K\\ i \end{array}\right)P_{F}^{i}{\left(1-P_{F}\right)}^{K-i},$$
  (37) $$P_{D_{0}}=\sum_{i=K_{\tau }}^{K}\left(\begin{array}{l} K\\ i \end{array}\right)P_{D}^{i}{\left(1-P_{D}\right)}^{K-i},$$
  *where* $K_{\tau }$ *is a discrete threshold.*
*(b)*  *For the Bayesian detection, the fusion error probability is given by*
  (38) $$P_{e}=P(H_{1})-P(H_{1})\sum_{i=T}^{K}\left(\begin{array}{l} K\\ i \end{array}\right)P_{D}^{i}{\left(1-P_{D}\right)}^{K-i}+P(H_{0})\sum_{i=T}^{K}\left(\begin{array}{l} K\\ i \end{array}\right)P_{F}^{i}{\left(1-P_{F}\right)}^{K-i}.$$
*The threshold T can be obtained as* $T=\left\lceil T^{*}\right\rceil$ *, where*
  (39) $$T^{*}=\frac{\ln\left\{\frac{P(H_{0})}{P(H_{1})}{\left(\frac{1-P_{F}}{1-P_{D}}\right)}^{K}\right\}}{\ln\left\{P_{D}(1-P_{F})/P_{F}(1-P_{D})\right\}}$$
  *and* ⌈⋅⌉ *denotes the standard ceiling function.*

**Proof.**  Under the condition that $L_{k}=L$ and $\sigma_{k}^{2}=\sigma^{2}$ for all *k*, we have $P_{D_{k}}=P_{D}$, $P_{F_{k}}=P_{F}$, and $\tau_{k}=\tau$. Define $K_{1}=\left|S_{1}\right|$, where $S_{1}=\left\{k:z_{k}>\tau \right\}$, i.e., $K_{1}$ is the cardinality of $S_{1}$. Thus, the TS fusion rule reduces to a simple counting rule:

(40) $$\Lambda_{TS}=K_{1}\ln\frac{P_{D}(1-P_{F})}{P_{F}(1-P_{D})}+K\ln\frac{\left(1-P_{D}\right)}{\left(1-P_{F}\right)}.$$

Note that the TS statistic is equivalent to $K_{1}$, which follows binomial distribution, i.e., $K_{1}|H_{1}\sim B(K,P_{D})$ and $K_{1}|H_{0}\sim B(K,P_{F})$. Given $K_{\tau }$, we obtain (36) and (37).

If the prior probabilities of $H_{0}$ and $H_{1}$ are known at the fusion center, we can employ the Bayesian approach to minimize the fusion error probability. Thus, we have

(41) $$\Lambda_{TS}\overset{H_{1}}{\underset{H_{0}}{≷}}\frac{P(H_{0})}{P(H_{1})}.$$

The fusion error probability can therefore be obtained as

(42) $$P_{e}=P(H_{0})P_{F_{0}}+P(H_{1})[1-P_{D_{0}}].$$

Substituting $P_{F_{0}}$ and $P_{D_{0}}$ into (42), we then obtain (38). Note that (39) can be derived by starting from (41) and exploiting the fact that the TS fusion statistic in Equation (40) is monotonic. □

### 4.3. Numerical Results

In this part, we show some simulation results. We consider the simplified scenario where sensors collaborate to detect a known parameter in Gaussian noise with zero mean and unitary variance. Each sensor makes its binary decision by employing a threshold test on the observation. Its false alarm rate is set to $P_{f}=0.05$ while the detection probability is $P_{d}=0.5$. The channel SNR is defined as ${\mathrm{SNR}}_{k}≜10\log_{10}(1/(2\sigma_{k}^{2}))$ dB. All the simulation results are obtained by averaging over $10^{5}$ realizations.

**Figure 2:**
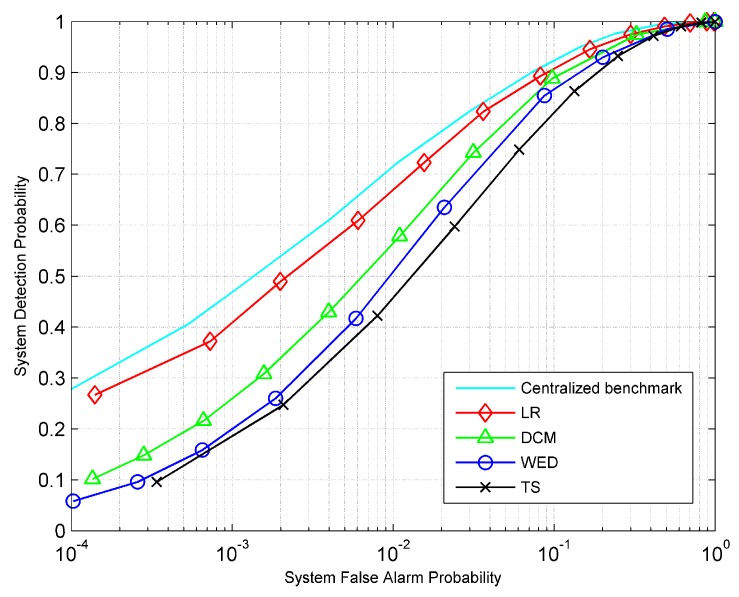
Receiver operating characteristic (ROC) curves for different fusion rules at *S* = 0 dB.

First, we consider the case where 30 sensors are partitioned into 10 groups, and different groups have different channel SNRs for their channels to the FC. Specifically, the sensor grouping strategy is denoted by $\left\{L_{k}\right\}=\left\{1,\;1,\;1,\;3,\;3,\;3,\;3,\;5,\;5,\;5\right\}$, respectively. We assume that the channel SNRs are distributed as $\left\{{\mathrm{SNR}}_{k}\right\}=\left\{S-6,\;S-4,\;S-2,\;S,\;S,\;S,\;S,\;S+2,\;S+4,\;S+6\right\}$ dB, respectively, where *S* is the arithmetic mean of all the channel SNRs. Figure 2 presents the receiver operating characteristic (ROC) curves for different fusion rules at *S* = 0 dB. Here, an idealistic centralized detection scenario, where sensors transmit their raw observations to the FC, serves as a performance baseline for distributed detection. As shown, the LR-based fusion rule performs closely to the centralized detection, and provides the optimal performance among all the distributed fusion rules. The DCM fusion rule is slightly worse than the optimal LR-based fusion rule and outperforms both WED and TS fusion rules.

**Figure 3:**
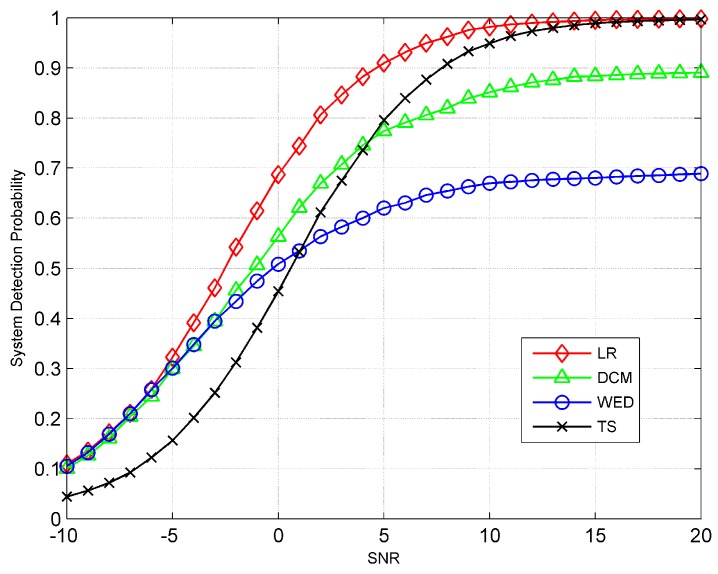
The probability of detection as a function of the mean value of channel SNRs for various fusion rules.

To better understand the performance difference as a function of channel SNR, Figure 3 gives the probability of detection as a function of the mean value of channel SNRs for various fusion rules. The system-level false alarm rate is fixed at $P_{F_{0}}=0.01$ (unless otherwise specified). From this figure, it is clear that the performances of WED and TS fusion rules approach that of the LR-based fusion rule at very low and very high SNR respectively. The simulation results confirm that the WED fusion rule is a low-SNR approximation of the LR-based fusion rule, and the TS fusion rule is a high-SNR approximation of the LR-based rule. However, when the channel SNR is medium, the DCM fusion rule has the best performance among the three sub-optimal rules. Table 1 gives the performance rating for the proposed fusion rules under different SNR range. In addition, we report the comparison of detection performance, computational complexity, and the requirement for a priori information for each of the fusion rules in Table 2.

**Figure 4:**
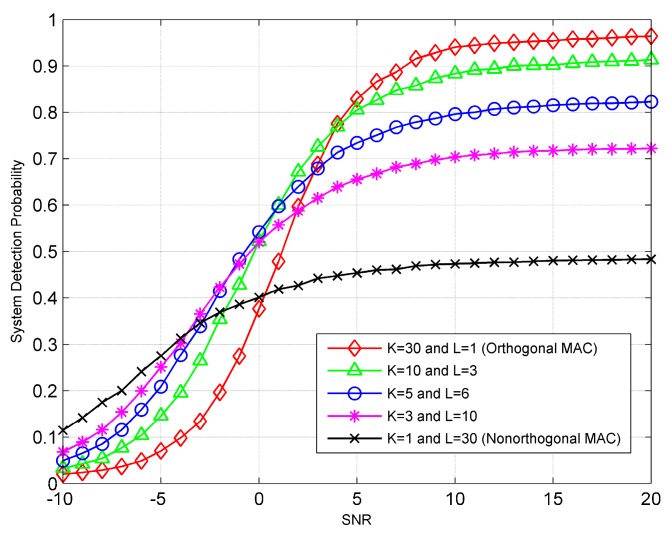
The probability of detection as a function of channel SNR for the ED fusion rule.

**Figure 5:**
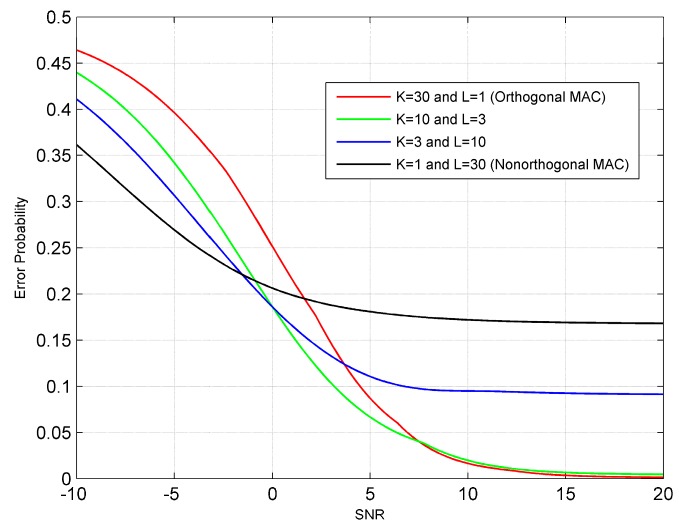
The error probability as a function of channel SNR for the TS fusion rule.

Next, we analyze the detection performance of the hybrid MAC under a uniform sensor grouping strategy. We assume that all the channels have the same SNR. Figure 4 gives the probability of detection as a function of channel SNR for the ED fusion rule. Figure 5 gives the numerically computed (i.e., using Equation (38)) error probability as a function of channel SNR for the TS fusion rule. Specifically, the prior probabilities of $H_{0}$ and $H_{1}$ are assumed to be equally likely. As shown in Figure 4 and Figure 5, the performance of the hybrid MAC is bounded by those of orthogonal and nonorthogonal MACs when the channel SNR is high or sufficiently low. In this case, the detection performances of both rules improve with the increase of group size when the channel SNR is low, and deteriorate with the increase of group size when the channel SNR is high. Note that, compared with the conventional orthogonal and non-orthogonal MACs, the hybrid MAC can provide a better ROC when the channel SNR is medium.

**Figure 6:**
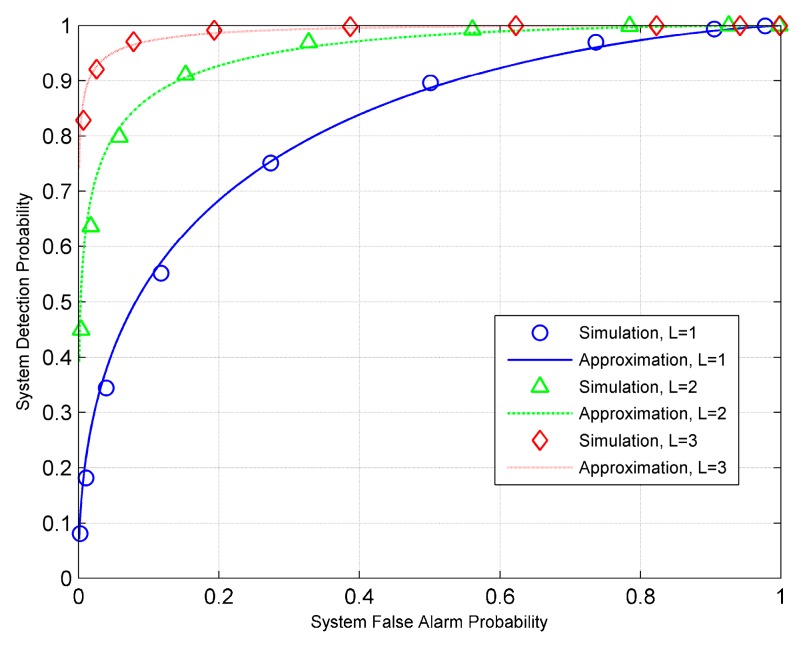
ROC curves for the ED fusion rule with *K* = 100.

**Figure 7:**
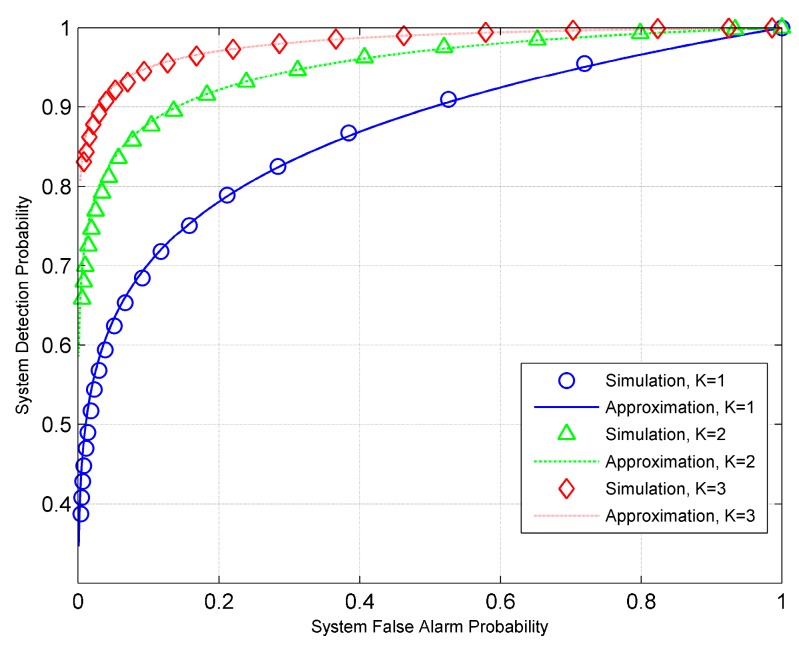
ROC curves for the ED fusion rule with *L* = 100.

Finally, we examine the quality of approximation for the ED fusion rule. Specifically, we set SNR=−5 dB. Figure 6 shows both the Gaussian approximation and the simulation results for a large number of groups. When the number of groups is 100, the Gaussian approximation is seen to provide a tight fit to the actual ROC for all values of *L* tested. We can also observe that increasing *L* results in a decrease in the gap between the approximation and simulation results. Figure 7 gives the ROC curves obtained by using the approximations (29) and (30), and those by simulations for *L* = 100. As shown, the approximation results agree very well with the simulation results. In particular, when *K* = 3, we see the approximation and simulation curves merging as shown in Figure 7.

## 5. Conclusions

In this work, we study the problem of decision fusion under the hybrid MAC scheme. Considering noncoherent detection at the fusion center, we present the optimal decision fusion rule based on the LR test and derive three sub-optimal rules with low-complexity. When sensors are uniformly grouped and all the channels have the same noise power, the WED and DCM fusion rules reduce to the ED fusion rule. For the ED rule, we derive closed-form results to the distribution of its fusion statistic under a large *K* and under a large *L*, respectively. In addition, we derive explicit formulas of the TS fusion statistic under the Neyman–Pearson criterion and the minimum probability of error criterion, respectively. Simulation results show that the WED fusion rule is a low-SNR approximation of the LR-based fusion rule, and the TS fusion rule is a high-SNR approximation of the LR-based fusion rule. The detection performance of the hybrid MAC generally improves with the increase of group size when the channel SNR is low, and deteriorates with the increase of group size when the channel SNR is high. Compared with the conventional orthogonal and nonorthogonal MACs, the hybrid MAC can achieve a better performance when the channel SNR is medium. In this work, we address only the fusion rule design for distributed detection of a known parameter in Gaussian noise. The detection of an unknown deterministic signal or a random time-correlated signal in noise is worth further investigation.

## Figures and Tables

**Table 1:** Performance rating under different SNR range.

Mean Value of Channel SNRs (dB)	Performance Rating
−10~−3	LR > WED > DCM > TS
−2~1	LR > DCM > WED > TS
2~4	LR > DCM > TS > WED
5~20	LR > TS > DCM > WED

**Table 2:** Comparison of the fusion rules.

Fusion Rule	Required Parameters	Complexity	Performance
LR	$P_{d}$, $P_{f}$, $\sigma_{k}^{2}$	Most complex	Optimum
WED	$P_{d}$, $P_{f}$, $\sigma_{k}^{2}$	Simple	Near-optimal for low SNR
DCM	$P_{f}$, $\sigma_{k}^{2}$	Most simple	Near-optimal for moderate SNR
TS	$P_{d}$, $P_{f}$, $\sigma_{k}^{2}$	Complex	Near-optimal for high SNR

## Appendix A

**Proof** **of Proposition 2.** Let Λ be a linear combination of ${z_{k}}^{\prime }s$. We have

(A1) $$\Lambda =\sum_{k=1}^{K}c_{k}z_{k},$$

where ${c_{k}}^{\prime }s$ denote the combining weights. Using (6) and algebraic manipulation, we have

(A2) $$E\left[\Lambda |H_{0}\right]=\sum_{k=1}^{K}c_{k}(L_{k}P_{f}+2\sigma_{k}^{2}),$$

(A3) $$E\left[\Lambda |H_{1}\right]=\sum_{k=1}^{K}c_{k}(L_{k}P_{d}+2\sigma_{k}^{2}),$$

(A4) $$\mathrm{Var}\left[\Lambda |H_{0}\right]=\sum_{k=1}^{K}c_{k}^{2}\left\{L_{k}^{2}P_{f}^{2}+L_{k}\left[(2+4\sigma_{k}^{2})P_{f}-2P_{f}^{2}\right]+4\sigma_{k}^{4}\right\}.$$

It immediately follows from (A2)–(A4) that

(A5) $$D(\Lambda )=\frac{{\left[\sum_{k=1}^{K}c_{k}L_{k}(P_{d}-P_{f})\right]}^{2}}{\sum_{k=1}^{K}c_{k}^{2}\left\{L_{k}^{2}P_{f}^{2}+L_{k}\left[(2+4\sigma_{k}^{2})P_{f}-2P_{f}^{2}\right]+4\sigma_{k}^{4}\right\}}.$$

Denote

(A6) $$a_{k}≜c_{k}\sqrt{L_{k}^{2}P_{f}^{2}+L_{k}\left[(2+4\sigma_{k}^{2})P_{f}-2P_{f}^{2}\right]+4\sigma_{k}^{4}}$$

and

(A7) $$b_{k}≜\frac{L_{k}(P_{d}-P_{f})}{\sqrt{L_{k}^{2}P_{f}^{2}+L_{k}\left[(2+4\sigma_{k}^{2})P_{f}-2P_{f}^{2}\right]+4\sigma_{k}^{4}}}.$$

Applying the Cauchy–Schwartz inequality, we have $D(\Lambda )\leq \sum_{k=1}^{K}b_{k}^{2}$, with the equality holding if and only if $a_{k}=\beta b_{k}$, where β is a constant. Thus, the combining weights in (A1) that maximize D(Λ) are given by

(A8) $$c_{k}=\beta \cdot \frac{L_{k}(P_{d}-P_{f})}{L_{k}^{2}P_{f}^{2}+L_{k}\left[(2+4\sigma_{k}^{2})P_{f}-2P_{f}^{2}\right]+4\sigma_{k}^{4}}.$$

Substituting (A8) into (A1) and neglecting the constant term that does not affect detection performance, we obtain the DCM fusion statistic as in (15). □
